# Quantifying the relative contributions of habitat modification and mammalian predators on landscape-scale declines of a threatened river specialist duck

**DOI:** 10.1371/journal.pone.0277820

**Published:** 2022-12-30

**Authors:** Amy L. Whitehead, John R. Leathwick, Douglas J. Booker, Angus R. McIntosh

**Affiliations:** 1 National Institute of Water and Atmospheric Research, Christchurch, New Zealand; 2 School of Biological Sciences, University of Canterbury, Christchurch, New Zealand; 3 Independent Conservation Science Consultant, Christchurch, New Zealand; University of Agriculture Faisalabad, PAKISTAN

## Abstract

Habitat modification and introduced mammalian predators are linked to global species extinctions and declines, but their relative influences can be uncertain, often making conservation management difficult. Using landscape-scale models, we quantified the relative impacts of habitat modification and mammalian predation on the range contraction of a threatened New Zealand riverine duck. We combined 38 years of whio (*Hymenolaimus malacorhynchos*) observations with national-scale environmental data to predict relative likelihood of occurrence (RLO) under two scenarios using bootstrapped boosted regression trees (BRT). Our models used training data from contemporary environments to predict the potential contemporary whio distribution across New Zealand riverscapes in the absence of introduced mammalian predators. Then, using estimates of environments prior to human arrival, we used the same models to hindcast potential pre-human whio distribution prior to widespread land clearance. Comparing RLO differences between potential pre-human, potential contemporary and observed contemporary distributions allowed us to assess the relative impacts of the two main drivers of decline; habitat modification and mammalian predation. Whio have undergone widespread catastrophic declines most likely linked to mammalian predation, with smaller declines due to habitat modification (range contractions of 95% and 37%, respectively). We also identified areas of potential contemporary habitat outside their current range that would be suitable for whio conservation if mammalian predator control could be implemented. Our approach presents a practical technique for estimating the relative importance of global change drivers in species declines and extinctions, as well as providing valuable information to improve conservation planning.

## Introduction

Habitat modification and introduced mammalian predators are two of the most important drivers of global extinctions and declines, particularly for avifauna on oceanic islands [1, 2]. However, the onset of these drivers is often highly correlated, with additive or synergistic effects likely [3, 4]. For example, Didham et al. [4, 5] argued that habitat modification is strongly correlated with both the introduction of mammalian predators and avian extinctions, making it difficult to disentangle the relative influence of these potential drivers of species declines [4, but see 6, 7]. These factors have led to considerable debate [e.g. 2–9], with little resolution over the relative influence of predation and habitat modification [but see 10, 11]. This limited understanding may result in uncertainty over whether to invest in predator control or habitat conservation/restoration, or whether both are essential for halting threatened species declines. Therefore, distingishing between these extinction drivers is important, both for understanding their relative influences in species declines and for improving conservation planning. Advances in species distribution modelling [e.g., 12, 13] and the availability of long-term, landscape-scale data now provide valuable tools to address this uncertainty.

The development of methods to derive high resolution measures of habitat at the scale of landscapes, coupled with the availability of large databases containing long-term species occurrence data, means it is now possible to use advanced statistical techniques to predict potential distributions over large areas [12]. Such techniques also enable hindcasting and forecasting of potential distributions under known or predicted past and future enviromental conditions [14–16]. Distribution data are often available for threatened species that have undergone considerable range contractions, providing opportunities to investigate the relative contributions of habitat modification and introduced predators to species declines [17]. Understanding the relative pressures on currently threatened species may provide valuable information to guide current conservation management and shed light on relative causes of past extinctions [5].

**Fig 1:**
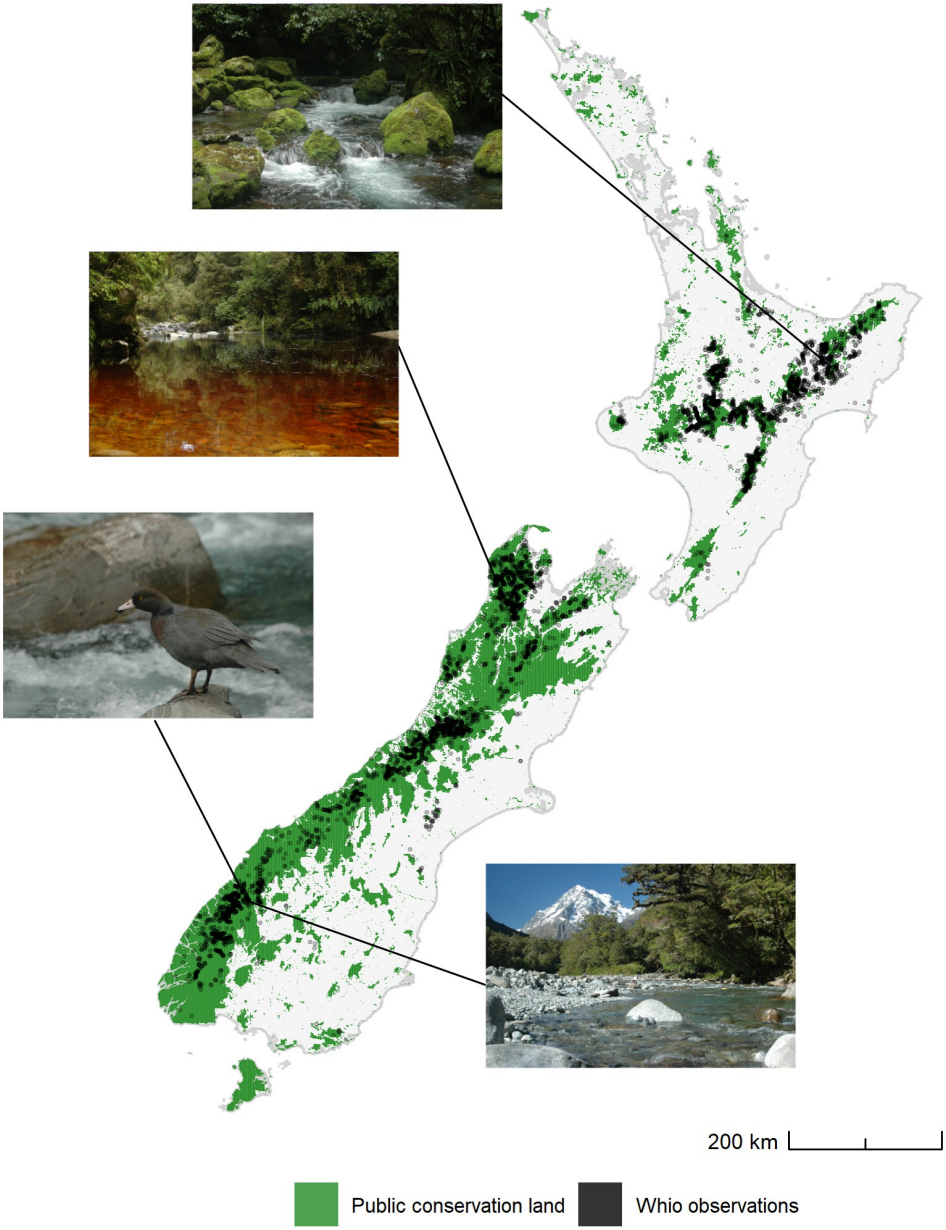
Distribution of whio observations in New Zealand from 1979–2016 relative to public conservation land. Sites where contemporary populations of whio occur show a high diversity of habitats, suggesting their range is not restricted by a lack of suitable habitat. Photos top to bottom: Whirinaki, Ōpārara, Cleddau, and Clinton Rivers [Photo credit–ALW]. Public conservation land republished from https://koordinates.com/layer/754/ under a CC BY license, with permission from Department of Conservation, original copyright 2017.

New Zealand’s avifauna has been severely depleted since human colonization, with habitat modification and introduced mammalian predators thought to be the primary drivers of extinction and decline for most species [18]. However, the relative impacts of these mechanisms on historic range contractions are unclear [11], making it difficult to prioritize and focus conservation management. We inform this debate by assessing the relative impacts of introduced predators and habitat modification on the decline of a threatened New Zealand duck. Whio (*Hymenolaimus malacorhynchos*, blue duck) are an endemic riverine duck predominantly now found on fast-flowing rivers in forested mountain catchments, where pairs actively defend a territory year round [19, 20]. They have declined in distribution and abundance due to habitat modification and predation by introduced mammals [21] and are currently listed as Nationally Vulnerable in the New Zealand Threat Classification system and Endangered on the IUCN Red List [22, 23]. New Zealand suffered severe habitat modification after human colonization by both Polynesian [ca 1280AD, 24, 25] and European settlers (1769AD), losing two-thirds of its native forest cover by the mid 20^th^ century [26]. Stoats (*Mustela erminea*), now considered the primary predators of whio [27], were introduced and became widespread in the late 1880s [28]. Therefore, initial contractions in whio range were likely due to a combination of habitat modification and predation by introduced mammals. Contemporary whio populations are sparsely distributed throughout New Zealand with small, fragmented populations in a wide range of unmodified habitats, largely protected within conservation reserves [Fig 1], [20, 29]. However, despite habitat protection, whio populations are still declining rapidly in the absence of predator control, with an estimated national population of less than 3,000 individuals [29–32]. Here, we use landscape-scale models to a) assess the relative influences of habitat modification and mammalian predation as potential drivers of historic whio range contractions and b) identify potential areas for prioritising whio conservation efforts.

## Methods

### Whio distribution data

Whio data were obtained from a New Zealand Department of Conservation (DOC) database containing 17,113 records of their occurrence in more than 166 river catchments across New Zealand. These data represent presence records collected from 1979–2016 during targeted surveys of river catchments looking for whio and routine monitoring of known whio populations, as well as incidental sightings by DOC staff and citizen scientists. Each whio occurrence record was buffered by 250 m and assigned to the nearest reach of the New Zealand digital river network, a representation of the spatial configuration of New Zealand’s rivers [33; version 1]; [Fig 1]. This network comprises ~576,000 reaches, each representing the length of river between confluences. The length of reaches in the network varies, but the average reach length is approximately 700 m. Where multiple whio occurrence records were assigned to the same reach, they were combined to give a single presence record to avoid pseudo-replication. In all, we identified 5,439 reaches where whio had been observed between January 1979 and January 2016. Given the large temporal and spatial coverage of the dataset, we have assumed that these reaches are representative of the true contemporary habitat of whio in New Zealand.

### Predictors

New Zealand has strong national-scale gradients in climate, geology, topography and hydrological regime. Each reach in the digital river network has been characterised by a set of predictors, including catchment area, stream length, elevation and slope derived from digital elevations models; catchment geology derived from geological maps; land cover from remote sensing data; and runoff, rainfall and potential evapotranspiration from interpolated climate station data [34]. These predictors have previously been used to predict the spatial distribution of invertebrate communities [35], freshwater fish species [36], availability of physical habitat [37, 38], hydrological indices [39], and hydraulic geometry characteristics [40] in rivers across New Zealand.

We selected predictors from the digital river network likely to be important in structuring whio distribution through physical habitat provision and food availability [20, 41, 42]. These predictors described characteristics of the hydrology, topography, climate and land cover for the entire New Zealand river network at two spatial scales ecologically relevant to whio; the local reach and upstream catchment (Table 1; further details in Leathwick *et al*. [43]). Whio are not restricted by downstream passage, so we did not consider the influence of downstream environmental conditions. In addition, we did not include a predictor describing the distribution of stoats, the primary predator of whio, because they are known to be ubiquitous across the New Zealand landscape [28, 44]. Similarly, due to a lack of appropriate national-scale data describing the distribution of predator control likely to benefit whio, we did not include the presence of predator control as a predictor.

**Table 1:** Predictors used in a boosted regression tree analysis to predict the relative likelihood of occurrence (RLO) for whio across New Zealand.

Category	Predictor	Whio median (range)	National median (range)
Climate	*segTempSummer–*Summer air temperature (°C).	15.17 (9.67 to 24.43)	16.29 (8.28 to 25.82)
	*segTempSeasonality–*Winter air temperature (°C), normalized with respect to summer air temperature $segTempSeasonality=\left(\left(\frac{W-\bar{W}}{\sigma_{W}}\right)-\left(\frac{S-\bar{S}}{\sigma_{S}}\right)\right)\times \sigma_{S}$ where *W* is the winter temperature for a reach, $\bar{W}$ is the average winter temperature for all segments, *σ*_*w*_ is the standard deviation of winter temperature, *S* is the summer temperature, and so on.	0.92 (-5.8 to 3.55)	0.39 (-7.2 to 5.25)
Topography	*segSinuosity–*Actual reach length divided by the Euclidian distance from the top to bottom of the reach (ratio). A measure of the sinuosity of a reach.	1.11 (1 to 3.79)	1.09 (1 to 8.89)
	*segSlope–*Mean segment slope (°).	0.03 (-0.05 to 0.97)	0.04 (-0.85 to 2.1)
	*segSlopeCatchment–*Mean local catchment slope (°).	8.09 (0 to 44.94)	6.28 (0 to 61.8)
Hydrology	*Feb–*Mean flow in February divided by mean flow over all time. Provides an estimate of flow seasonality (ratio).	0.75 (0.42 to 1.67)	0.59 (0.22 to 1.71)
	*FRE3 –*Average number of events per year that exceed three times the median flow (events/year).	17.48 (4.04 to 35.85)	13.05 (1.81 to 40.94)
	*segFlowStability–*Annual low flow/annual mean flow (ratio).	0.3 (0.01 to 0.59)	0.16 (-0.1 to 0.63)
	*MeanFlow–*Mean flow over all time (m^3^ s^-1^).	1.12 (0 to 309.59)	0.03 (0 to 1327.78)
Stream size	*Order–*An ordinal measure of the relative size of streams (1–8).	3 (1 to 7)	1 (1 to 8)
	*WidthQ50 –*Mean wetted width at median flow (m).	7.37 (0.27 to 101.81)	1.18 (0.01 to 136.11)
Habitat	*segIndigenousForest–*Proportion of local catchment with indigenous vegetation.	0.90 (0 to 1)	0.00 (0 to 1)
	*usIndigenousForest–*Proportion of upstream catchment with indigenous vegetation.	0.57 (0 to 1)	0.01 (0 to 1)
	*segShade–*Riparian shade (proportion).	0.72 (0 to 0.8)	0.58 (0 to 0.8)
	seg*Habitat*–Weighted average of proportional cover of local habitat using categories of 1–still; 2–backwater; 3–pool; 4–run; 5–riffle; 6–rapid; 7–cascade.	4.34 (3.24 to 6.52)	4.03 (1 to 6.9)
	*segSediment–*Weighted average of proportional cover of bed sediment using ordinal categories of 1–mud; 2–sand; 3–fine gravel; 4–coarse gravel; 5–cobble; 6–boulder; 7–bedrock.	4.66 (1.45 to 6.31)	3.94 (1 to 6.79)
	*segGravelCobble*–Predicted proportion of bed sediment that is gravel or cobble (proportion).	0.55 (0.18 to 0.75)	0.52 (0.14 to 0.83)

Predictors were obtained from the Freshwater Ecosystems of New Zealand (FENZ) geodatabase, linked to the New Zealand digital river network [34]. Values represent the median (and range) for each predictor at reaches where whio were present and for the entire New Zealand river network under contemporary conditions. See S1 File for graphical comparisons of contemporary and pre-human habitat conditions.

### Statistical modelling

Species distribution modelling requires knowledge about the presence and absence of the species of interest to allow the model to evaluate habitat occupancy. Our whio dataset did not contain absence data, so we applied the method of Barbet-Massin et al. [45] to use equal numbers of presence and background points for machine-learning techniques. We randomly selected 5,439 reaches from a geographically constrained area based on a kernel density plot of whio presences to use as background points where whio were assumed to be absent. Restricting the area for selection of background points prevents artificial inflation of the test statistics, producing a more realistic measure of important predictors [46–48].

**Fig 2:**
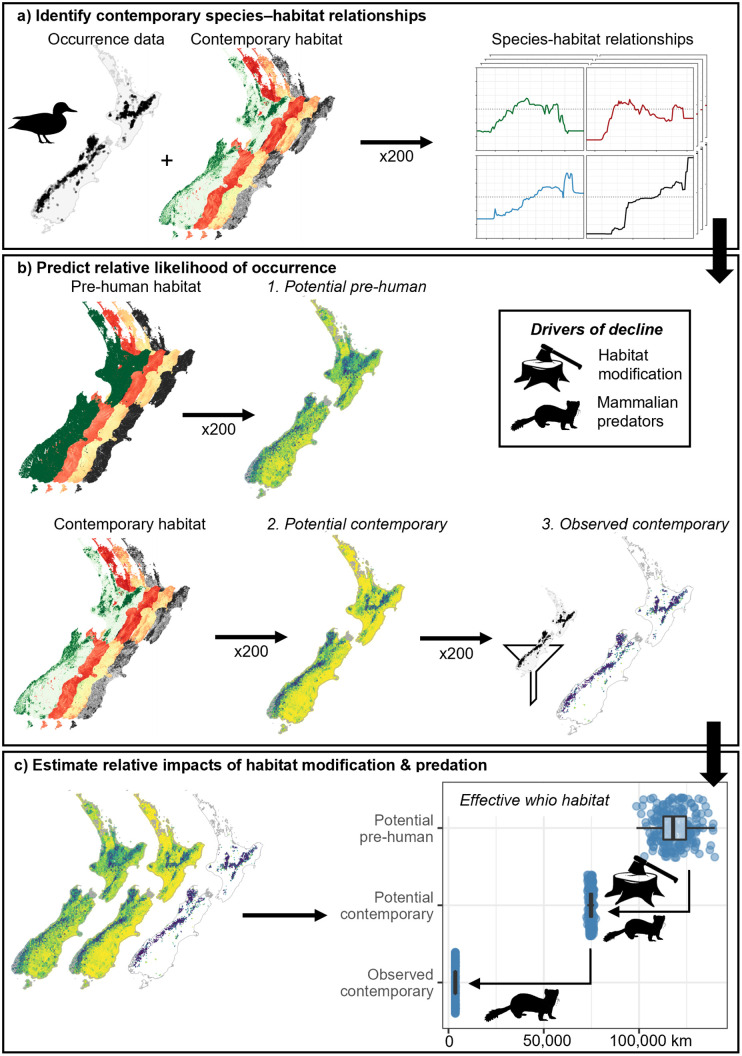
The approach used to assess the relative impacts of habitat loss and mammalian predation on whio range declines. a). Whio occurrence and contemporary habitat data were combined in 200 bootstrapped boosted regression tree (BRT) models to identify species-habitat relationships. b). We used these relationships to predict the relative likelihood of occurrence (RLO) of whio under two scenarios: *potential pre-human* with pre-human land cover and no mammalian predators; and *potential contemporary* with contemporary land cover and no mammalian predators. The *observed contemporary* results filtered the potential contemporary RLO to known whio occurrence sites only, assuming mammalian predators occur elsewhere. c). Finally, we estimated the relative impact of habitat loss and mammalian predation on whio range declines by comparing the effective whio habitat (sum of RLO x reach length, km) between scenarios.

We developed models of the relative likelihood of occurrence (RLO) for whio using boosted regression trees [BRT, 49] (Fig 2a). BRT models incorporate the advantages of two statistical techniques: regression trees (models that relate a response to their predictors by recursive binary splits) and boosting (an adaptive method for combining many simple models to give improved predictive performance) [50]. BRT models are capable of dealing with non-linear relationships between predictors and can assess high-order interactions, making them particularly suited for ecological data [50]. BRT models are also robust to the effects of outliers and irrelevant predictors [51] and have previously been used to model species distributions [e.g., 12] and ecosystem responses to regional gradients of stressors [52].

All analyses were carried out in R [53; Version 3.3.3] using the ‘dismo’ package [13] and functions adapted from Elith et al. [50]. Our initial BRT model used the full dataset and was allowed to fit interactions, using a tree complexity of five and a learning rate of 0.01 [see 50 for definitions of these parameters].

As BRT models have be known to over-fit the training data [50], we used ten-fold cross validation to determine the optimal number of trees, and to assess model performance. Two values were calculated to assess model performance: the predictive deviance [50], and the discrimination between presences and absences as measured by the area under the receiver operator characteristic curve [AUC; 54]. High AUC values occur when areas of known presences are predicted to have a high RLO and areas of known (or assumed) absences have a low predicted RLO, while an AUC value of 0.5 indicates a model that predicts no better than random. Finally, we ran 200 model simulations, each fitted to a bootstrapped re-sample of the original dataset that allowed us to calculate uncertainty estimates around the predicted RLOs (Fig 2a). These bootstrapped simulations each used the model structure of the full model but randomly withheld 20% of the data from the full dataset. The withheld data were used to assess model performance as described above. We provide detailed model descriptions in S2 File following the ODMAP protocol [55].

### Spatial predictions to quantify drivers of decline

After fitting, the bootstrapped BRT models were used to generate spatial predictions of whio RLO across the digital river network for each simulation under two different scenarios (Fig 2b). The first scenario represented the predicted RLO using reach and catchment predictors that describe the present-day environment. Here we assumed that reaches with a high RLO would be suitable whio habitat if mammalian predators, particularly stoats, are able to be controlled (referred to as the *potential contemporary* distribution). Under this scenario, we also evaluated the predictions at reaches where whio had been observed (*observed contemporary*), assuming these reaches reflect the true contemporary distribution of whio and whio are absent from all other reaches. Here we were interested in comparing the predicted RLO at known whio locations to the predicted contemporary RLO across the entire New Zealand river network to assess the potential loss of habitat due to mammalian predators. The second scenario represented the predicted RLO with land cover prior to anthropogenic alteration and in the absence of mammalian predators. For this scenario, spatial predictions of the *potential pre-human* distribution of whio were derived by replacing the contemporary values of *segIndigenousForest*, *usIndigenousForest*, *segShade*, *segTempSummer* and *segTempSeasonality*, with values estimated for each reach prior to human arrival in New Zealand [S1 File], [56]. Here, we assumed that reaches with a high RLO would be suitable whio habitat under conditions of pre-human land cover and in the absence of mammalian predators. For each scenario, we calculated the mean (± sd) RLO for each reach across all bootstrapped simulations to estimate the potential spatial distribution of whio. Spatial patterns in prediction uncertainty for each scenario were characterized by examining the coefficient of variation of predictions across the 200 bootstrap simulations at each reach. We also assessed the degree to which the model predictions could be extrapolating into novel environmental space under each scenario using the extrapolation detection (ExDet) tool [57, 58]. ExDet compares the range of environmental predictors at reference sites (whio occurrences) to those available across the landscape to detect and quantify the degree of dissimilarity for points that are either outside the univariate range or form novel covariate combinations (correlations) but were still within the univariate range.

To estimate the amount of potentially available habitat under each scenario for each simulation, we multiplied the length of each reach by its RLO to calculate an index of *effective whio habitat* and summed this across the entire river network. By comparing the predicted potential distributions with the observed contemporary distribution of whio (as represented by the predicted RLO under the potential contemporary scenario at the reaches where whio were observed from 1979–2016), we estimated the potential relative impacts of habitat modification and mammalian predation on whio measured in terms of potential river length occupied (Fig 2c). The impact of habitat modification was evaluated by calculating the reduction in effective whio habitat from pre-human conditions compared to the potential contemporary distribution in the absence of predators. We then assumed that the difference in effective whio habitat between the potential contemporary distribution and observed contemporary distribution represented the likely impacts of mammalian predators on whio.

## Results

Whio observations were widely distributed across New Zealand, occupying approximately 4,400 linear km of riverine habitat across their known range and types of habitat they typically occupy [20, 29, 41]. They were most frequently observed in small to medium forested mountain streams, with 75% of occupied reaches in relatively unmodified environments within protected conservation reserves (Table 1, Fig 1).

**Fig 3:**
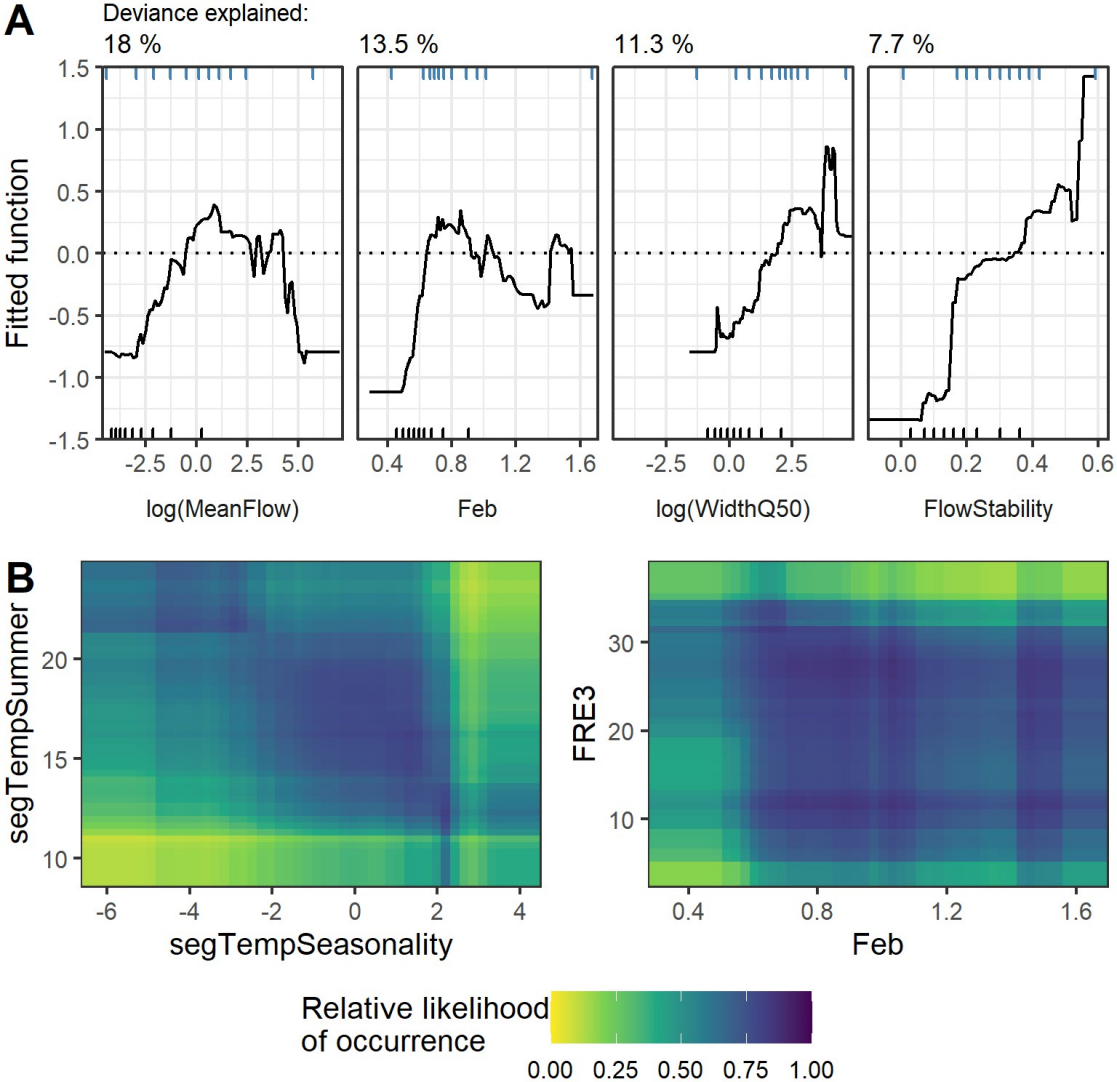
Key relationships between habitat and the relative likelihood of occurrence (RLO) for whio. (A) The four most important predictors in the full boosted regression tree (BRT) model. The dotted line represents the threshold above which whio occurrence is likely. Decile ticks at the bottom of each plot show how the contemporary New Zealand riverine environment is distributed for each predictor, while decile ticks at the top of each plot show how whio are distributed. (B) Partial dependence plots for the two strongest interactions in the full BRT model of whio occurrence, with darker colors indicating environmental space where whio are mostly likely to occur. To produce the plots, predictors except those plotted were held at their means. See Table 1 for predictor descriptions and units.

Bootstrapping indicated that our BRT model had excellent discriminatory power (AUC = 0.925 ± 0.000; S1 Table) and explained 69.4 ± 0.2% of the model deviance. The models showed that the contemporary distribution of whio is characterized by a combination of local environmental conditions and factors operating in the upstream catchment (Fig 3, Table 2). Whio were predicted to most likely occur in small to medium-sized rivers with stable flows, where the local and upstream catchments were characterized by a high proportion of indigenous forest (Fig 3a). We also identified a strong interaction between the reach-scale summer temperature and temperature seasonality, with the RLO for whio increasing in reaches with lower winter temperatures relative to their summer temperatures (Fig 3b).

**Table 2:** Important predictors averaged across 200 bootstrapped boosted regression tree (BRT) models predicting the relative likelihood of occurrence (RLO) for whio across New Zealand.

a) Relative contribution of predictors	
Predictors	Relative Contribution (%)
log(MeanFlow)	15.6 ± 2.5
Feb	13.2 ± 1.6
log(WidthQ50)	11.7 ± 2.4
segFlowStability	7.7 ± 0.6
segIndigenousForest	7.3 ± 1.0
FRE3	6.0 ± 0.4
segSediment	5.8 ± 0.6
usIndigenousForest	5.5 ± 0.7
segTempSeasonality	4.8 ± 0.3
segShade	4.1 ± 0.6
segTempSummer	4.0 ± 0.3
segGravelCobble	3.2 ± 0.2
segSlopeCatchment	2.8 ± 0.2
segSlope	2.7 ± 0.2
log(segSinuosity)	2.6 ± 0.2
segHabitat	2.6 ± 0.2
Order	0.5 ± 0.1
b) Pairwise interactions between predictors	
Predictor combination	Interaction strength
segTempSeasonality & segTempSummmer	173.8 ± 47.1
Feb & FRE3	52.8 ± 28.5
segTempSummmer & FRE3	34.8 ± 13.5
segSediment & segTempSeasonality	31.6 ± 13.7
Feb & segFlowStability	30.6 ± 9.6

a) Mean (± sd) relative contribution of the predictors. b) The top five pairwise interactions between predictors. The interaction strength indicates the relative degree of departure from a purely additive effect, with a value of zero indicating no interaction. See Table 1 for predictor descriptions and units.

**Fig 4:**
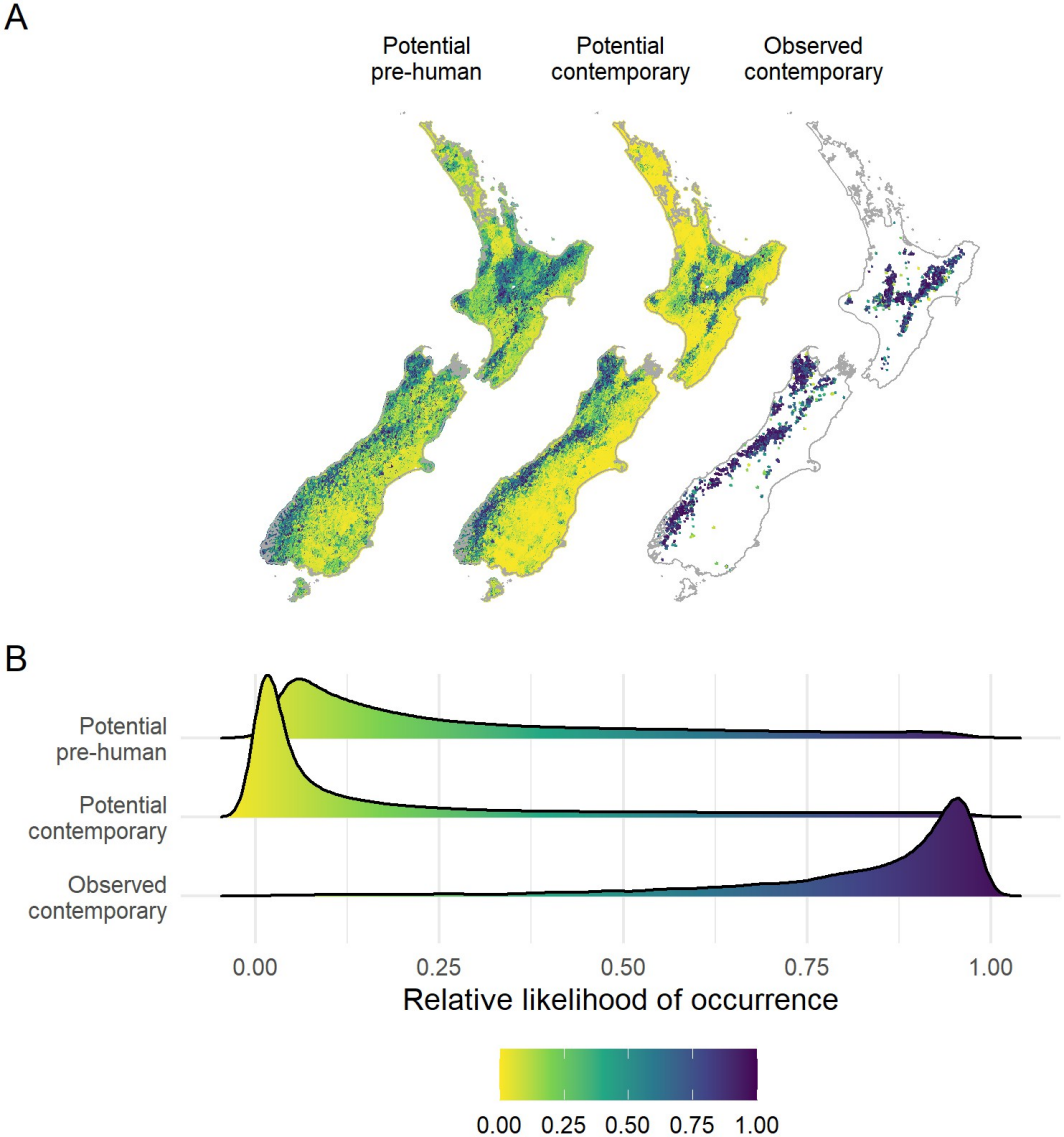
Predicted relative likelihood of occurrence for whio under three scenarios. A) Spatial distribution and B) density plots of the mean predicted relative likelihood of occurrence for whio prior to human arrival in New Zealand (potential pre-human), in contemporary habitat with the exclusion of predators (potential contemporary) and the observed distribution (observed contemporary). Potential values represent predictions from 200 bootstrapped simulations of a boosted regression tree model, while observed values are the potential contemporary predictions at reaches where whio were observed from 1979–2016.

The predicted RLO for whio under the potential contemporary scenario reflects spatial patterns in the dominant environmental predictors (Fig 4), with higher values in mountainous regions dominanted by indigenous forest and small to medium-sized, stable rivers. The mean RLO of reaches in the observed contemporary reaches (0.82 ± 0.19) was high relative to the full river network under contemporary conditions (0.18 ± 0.25). RLO values increased in 82% of reaches under the potential pre-human scenario, with an average increase of 0.11 ± 0.16 (range: -0.65 to 0.81). The spatial distribution of RLO differed between the scenarios, with RLO highest in more mountainous and forested terrain under the potential contemporary scenario but more widespread in the potential pre-human scenario. The models were extrapolating into novel environmental space across 17.2% and 18.1% of the river network under the potential contemporary and potential pre-human scenarios, respectively (S1 Fig). Only 0.01% of this extrapolation was driven by novel combinations of predictors, with the remainder caused by univariate predictors being beyond the known range of whio occurrences (Table 1). The most influential predictors contributing to extrapolation under both scenarios were *Feb* and *segHabitat* (S2 Fig), with the greatest extrapolation occurring in areas with relatively drier summers or more stable, slower flowing rivers. On average, mean RLO was lower in areas where univariate extrapolation was identified under both scenarios.

On average, prediction uncertainty was highest under the potential pre-human scenario (0.31 ± 0.13) compared to the potential contemporary scenario (0.25 ± 0.11), with a higher coefficient of variation recorded at 72.2% of reaches (S3 Fig). Low prediction uncertainty was noted in observed contemporary reaches (0.07 ± 0.08) compared to the full river network under contemporary conditions. Uncertainty in the predictions was highest at intermediate RLO values, with some evidence of spatial patterning in prediction uncertainty (S3 Fig).

**Fig 5:**
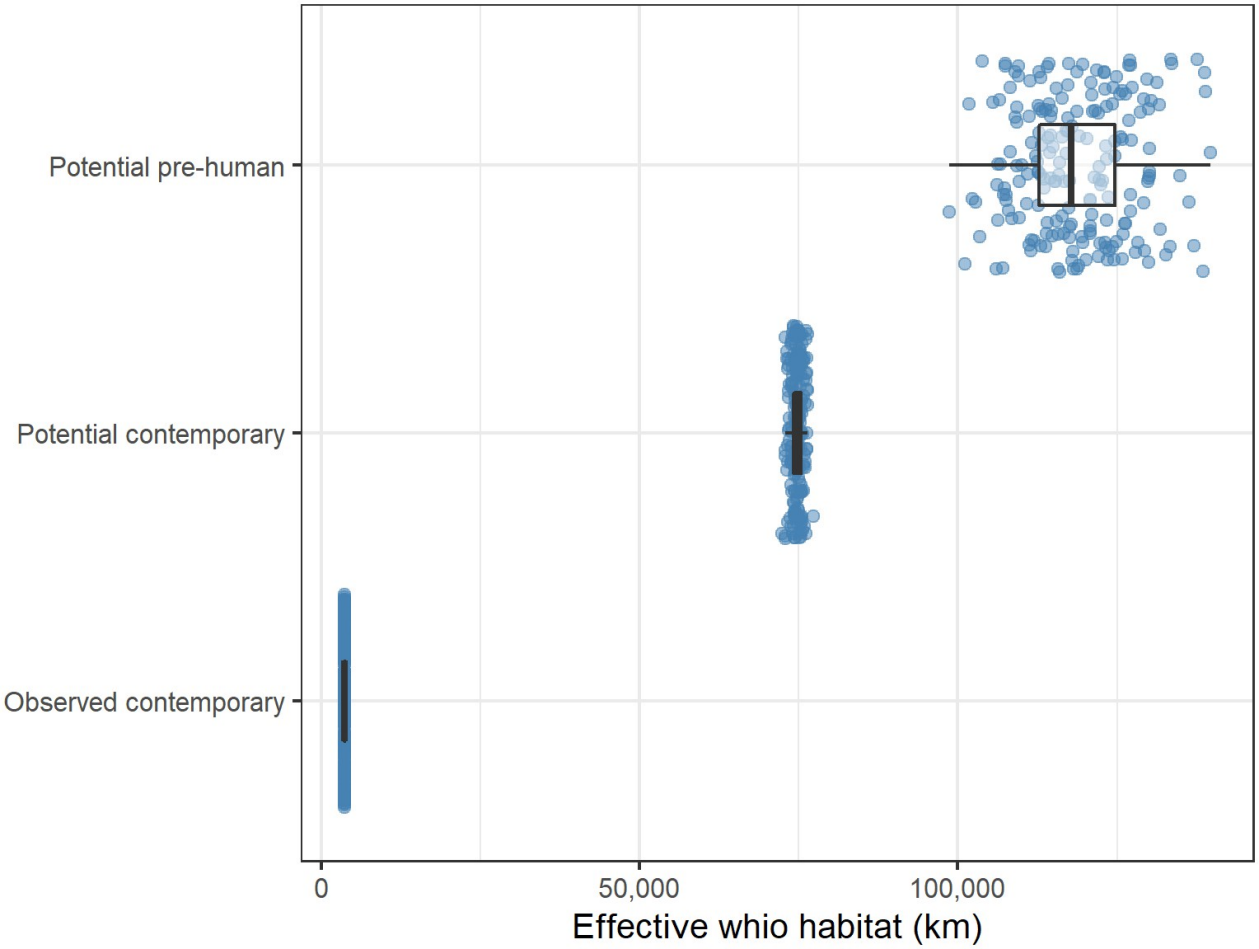
Availability of predicted effective whio habitat under three scenarios. Effective whio habitat is the sum of the predicted relative likelihood of occurrence for each reach x reach length prior to human arrival in New Zealand (potential pre-human), in contemporary habitat with the exclusion of predators (potential contemporary) and the observed distribution (observed contemporary). Potential values represent predictions from 200 bootstrapped simulations of a boosted regression tree model, while observed contemporary values are the potential contemporary predictions at reaches where whio were observed from 1979–2016. Boxplots show the median (solid line) and quartiles with whiskers reaching up to 1.5 times the interquartile range.

The predicted observed contemporary populations of whio occupy approximately 3,597 ± 16 km of effective whio habitat (sum of RLO x reach length; Fig 5). Based on the contemporary environmental data, our models predicted that there are 74,736 ± 857 km of effective whio habitat available if we could exclude mammalian predators. In comparison, we estimated that 118,740 ± 8,531 km of effective whio habitat was potentially available prior to human arrival in New Zealand. A large proportion of the predicted pre-human range, particularly in the North Island and eastern South Island, occurred in areas subsequently cleared of native vegetation after human arrival (Fig 4, S1 File). Thus, we estimate that historic habitat modification, combined with mammalian predation, reduced effective whio habitat by 36.7 ± 4.5%. Habitat within the predicted potential contemporary range of whio is largely unmodified, meaning that effects of mammalian predators are the likely cause of an additional 95.2 ± 0.05% contraction in their range.

## Discussion

By revealing the magnitude of population declines and partitioning the mechanisms, our analyses identify mammalian predation as the major driver of whio range contraction. They also provide guidance to managers by highlighting the large amount of suitable habitat that whio could potentially occupy if the effects of predation can be mitigated. Controlling stoats is possible, with large-scale, low-intensity predator control significantly increasing whio productivity and population viability [27, 30, 32]. Currently over 1,550 km of contemporary whio habitat (or 1.5% of their likely pre-human range) is actively managed [59]. However, conservation managers have considerable scope to expand whio populations into new areas using predator control and could prioritize sites based on our predictions of the RLO. Indeed, whio populations are already benefiting from an expansion of predator control networks by both government agencies and local community groups [30, 32, 59]. To help facilitate prioritisation of future conservation efforts for whio, we have made the predictions of mean RLO for whio under contemporary habitat conditions available via the NZ River Maps webtool (https://shiny.niwa.co.nz/nzrivermaps), an online platform for visualising environmental data on the New Zealand digital river network [60, 61].

Explicitly considering the relative effects of drivers of species decline is vital for understanding past declines and developing effective conservation management. Introduced mammalian predators have long been recognized as a contributing factor in the decline of avian species, particularly on oceanic islands [1, 62, 63], but their relative influence compared to other drivers has been debated [2–10]. Our approach provides a method of quantifying the relative effects of multiple mechanisms of species decline and extinction by using landscape-scale modelling techniques to analyse readily available information about species distributions. We have shown that predation by introduced mammals is the primary mechanism driving historic whio declines, supporting previous research based on contemporary populations [27, 30–32]. Indeed, several populations within our occurrence dataset are known to have gone locally extinct between 1979–2016 (e.g., Catlins River), despite no significant habitat modification. However, our analysis also highlights the likely combined effects of mammalian predation and habitat modification. It is difficult to establish how these drivers interacted because there are few historical whio observations and the period of widespread habitat modification was correlated with the introduction of mammalian predators. However, habitat modification likely played a role in the early declines and localized extinctions of whio populations and may have excaerbated the impact of mammalian predators [64, 65]. For example, low resource availiability in modified habitats (e.g., different prey communities, limited nesting habitat) may have resulted in lower population growth rates, making populations more susceptible to predation. In addition, we cannot rule out the contribution of other factors, such as demographic stochasticity, disease, competition from non-natives species (e.g, salmonid fishes), climate change and genetic and Allee effects, to population declines but it is most likely that these would have only compounded the effects of predation [8, 11, 66]. Indeed, there is little evidence to suggest that such factors play an important role in structuring the distribution or demographic stability of contemporary whio populations [67]. Introduced predators are recognized as the greatest threat to contemporary island avifauna [2, 11] and our findings support previous research suggesting that they were a major factor in avian extinctions on oceanic islands [2, 8, 10, 62, 63].

Although boostrapping showed that uncertainty in our models was very low, the accuracy of predictions from species distribution models, particularly when predicting to novel environments, is influenced by the availability of suitable data to parameterise the models, the model structure and the similarity of the environmental space in the training and prediction datasets [68]. The need to extrapolate into novel environmental space may occur if the species occupies a narrow range of habitats compared to the overall environment [57]. Testing for this issue indicated that our models were extrapolating beyond our training data when predicting to around 18% of the New Zealand river network, predominantly in places where one or more environmental predictors were outside the known contemporary range of whio for both the pre-human and contemporary datasets. It is possible that these areas of extrapolation represented marginal pre-human habitat for whio along the edge of their natural range, with higher probabilities of local extirpation due to lower population densities [69]. Therefore, populations in these areas may have faced more rapid declines in response to both habitat modification and mammalian predation. However, given the limited information about the actual historic range of whio, it is difficult to know whether the environmental gradients that whio currently occupy are truly reflective of pre-human habitat.

In addition to novel environments outside the training data, model predictions may not accurately reflect habitat suitability if spatial patterns of habitat use are not related to habitat quality [70] or when observations are temporally biased with respect to changing habitats [71]. Such patterns of habitat use may result from environmental changes removing high quality habitats, making them inaccessible to individuals, or an increase in the attractiveness of low quality habitats despite reductions in population viability [e.g., ecological traps, 72, 73]. Temporal mis-matches may result if the habitat predictors used to develop SDMs do not align with the period in which the observations occurred. The whio occurrence dataset used to develop our BRT models included observations from 1979–2016 and is thought to characterize a wide range of contemporary habitats and be representative of their known range. In addition, the most important predictors identified by our model align with previous research, indicating that climatic and instream hydraulic conditions have an important influence on the distribution of whio Collier *et al*. [20]. While it is possible that these contemporary habitats do not accurately reflect the preferred pre-human habitat conditions of whio, there is currently no evidence to suggest that this is the case. Therefore, it seems unlikely that these issues will have had a strong effect on our ability to distinguish between the drivers of decline.

The increasing vulnerability of species to global change, coupled with a limited resource pool for conservation, means that effective conservation management is critical for species persistence. Understanding the mechanisms of global change that drive species declines will help us to improve the focus of management towards appropriate threats, particularly in these times of rapid anthropogenic change. Our approach presents a practical technique for estimating the relative importance of global change drivers in species declines and extinctions. In addition, the outputs could provide valuable information to inform conservation planning by identifying both the primary driver(s) of decline and locations where actions to manage these drivers might best be targeted for maximum conservation benefit [74].

## Supporting information

S1 File Graphical representation of changes between pre-human and contemporary habitat for five predictors across the New Zealand river network. These data were used to predict the potential pre-human and potential contemporary distribution of whio, respectively.
(PDF)

S2 File ODMAP (Overview, Data, Model, Assessment and Prediction) protocol describing the species distribution modelling approach used in this analysis using a standardized reporting framework. (PDF)

S1 Table Mean (± se) predictive performance of a boosted regression tree (BRT) model predicting the relative likelihood of occurrence (RLO) for whio across New Zealand. Performance of the full model was assessed using 10-fold cross-validation, while performance of the 200 bootstrapped models was assessed by comparing predictions to data withheld during each simulation.
(PDF)

S1 Fig The areas where our boosted regression tree models will be extrapolating within the pre-human and contemporary environments with respect to whio occurrences. Negative values (purple) indicate areas with at least one predictor outside the univariate range of the whio occurrences (Table 1). Values greater that one (orange) indicates areas that are within the univariate coverage of the whio occurrences but represent non-analogous predictor combinations. Values ranging 0 to 1 (green) are similar to the whio occurrences as they both fall within the range of predictors and capture the same predictor combinations.
(TIF)

S2 Fig Predictors contributing to univariate extrapolation. A) The most influential univariate predictors affecting extrapolation within the pre-human and contemporary environments with respect to whio occurrences. B) The spatial distribution of the most dissimilar predictor relative to whio occurrences at a given location. Predictors contributing less than 1.5% to univariate extrapolation have been combined into *Other*, while *None* reflects areas where no predictor is outside the coverage of the whio occurrences or with non-analogous combinations. Note that *segIndigenousForest* and *usIndigenousForest* do not contribute to extrapolation as whio occur across the full range of values in the environment (Table 1). See Table 1 for predictor description and units.
(TIF)

S3 Fig A) Spatial distribution and B) density plots of uncertainty (coefficient of variation) in the relative likelihood of occurrence (RLO) prior to human arrival in New Zealand (potential pre-human), in contemporary habitat with the exclusion of predators (potential contemporary) and the observed distribution between 1979 and 2016 (observed contemporary). The potential values are based on predictions from 200 bootstrapped simulations of a boosted regression tree model, while the observed values represent the uncertainty from the potential contemporary models at all reaches where whio were known to occur between 1979 and 2016. The top 2.5% of uncertainty values have been removed to add visual interpretation of the data.
(TIF)

S4 Fig The difference between the mean and coefficient of variation of predictions under the potential pre-human and potential contemporary scenarios. Negative values (purple) indicate areas with lower pre-human values, while positive values (green) occur in areas with higher pre-human values.
(TIF)
