# Supplementary material for: Quantifying the relative contributions of habitat modification and mammalian predators on landscape-scale declines of a threatened river specialist duck
Source: PLoS One. 2022 Dec 30;17(12):e0277820. doi: 10.1371/journal.pone.0277820 (PMC9803212; doi:10.1371/journal.pone.0277820)
Supplement: S1 File — These data were used to predict the potential pre-human and potential contemporary distribution of whio, respectively. (PDF) [file pone.0277820.s001.pdf]

S1 File. Graphical representation of changes between pre-human and contemporary habitat for five predictors across the New Zealand river network. These data were used to predict the potential pre-human and potential contemporary distribution of whio, respectively.

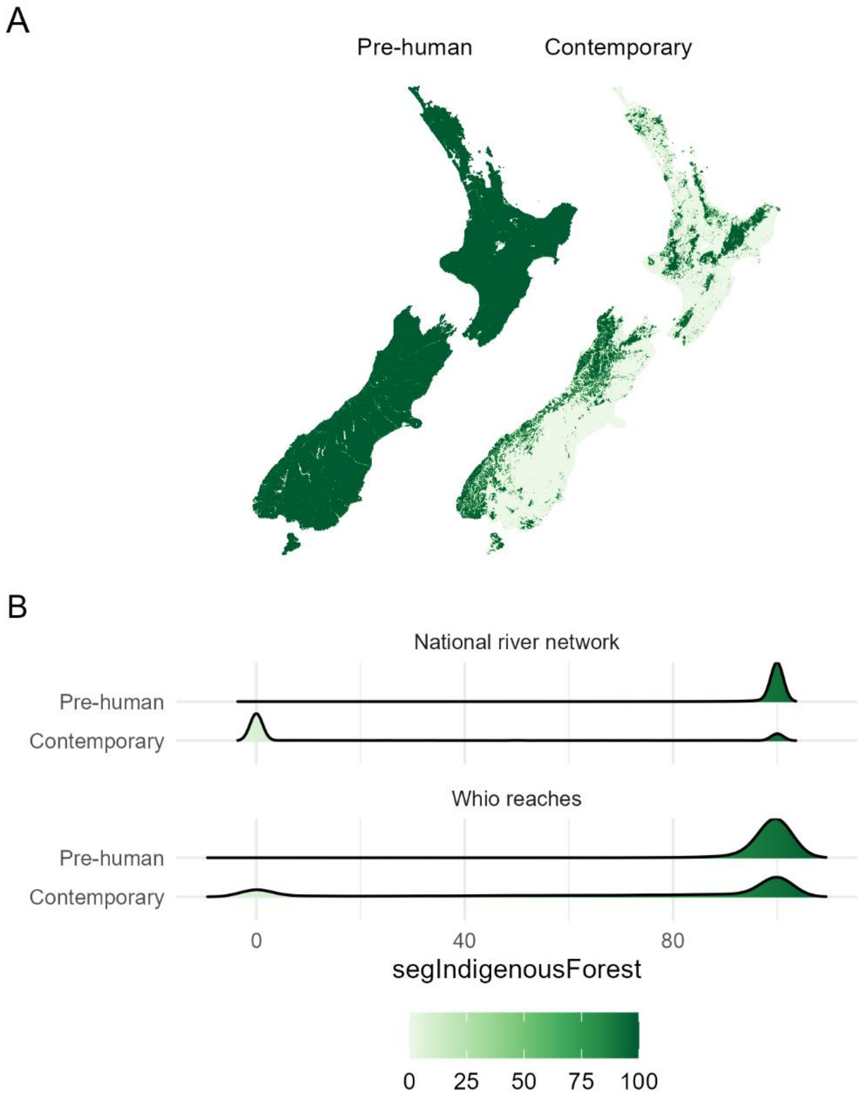

S1 Fig A. A) Spatial distribution and B) density plots of pre-human and contemporary indigenous forest cover (*segIndigenousForest*) across the national river network and at reaches where whio are known to occur. These data were used to predict the potential pre-human and potential contemporary distribution of whio, respectively. See Table 1 for predictor description and units. Pre-human indigenous forest cover was derived from the Potential vegetation of New Zealand spatial layer (republished under a CC BY license with permission from Landcare Research New Zealand Limited, original copyright 2012), while contemporary indigenous forest cover was obtained from the Freshwater Ecosystems of New Zealand (FENZ) geodatabase (republished under a CC BY license, with permission from Department of Conservation, original copyright 2010).

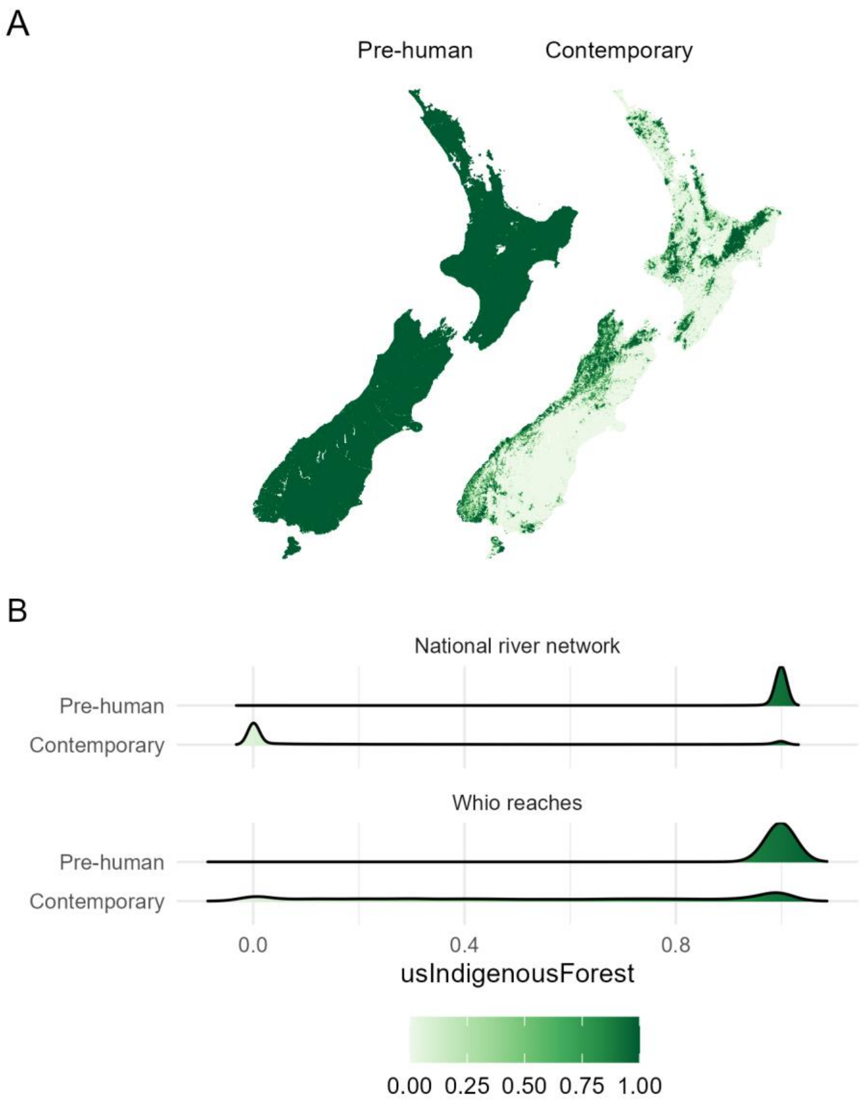

S1 Fig B. A) Spatial distribution and B) density plots of pre-human and contemporary upstream indigenous forest cover (*usIndigenousForest*) across the national river network and at reaches where whio are known to occur. These data were used to predict the potential pre-human and potential contemporary distribution of whio, respectively. See Table 1 for predictor description and units. Pre-human indigenous forest cover was derived from the Potential vegetation of New Zealand spatial layer (republished under a CC BY license with permission from Landcare Research New Zealand Limited, original copyright 2012), while contemporary indigenous forest cover was obtained from the Freshwater Ecosystems of New Zealand (FENZ) geodatabase (republished under a CC BY license, with permission from Department of Conservation, original copyright 2010).

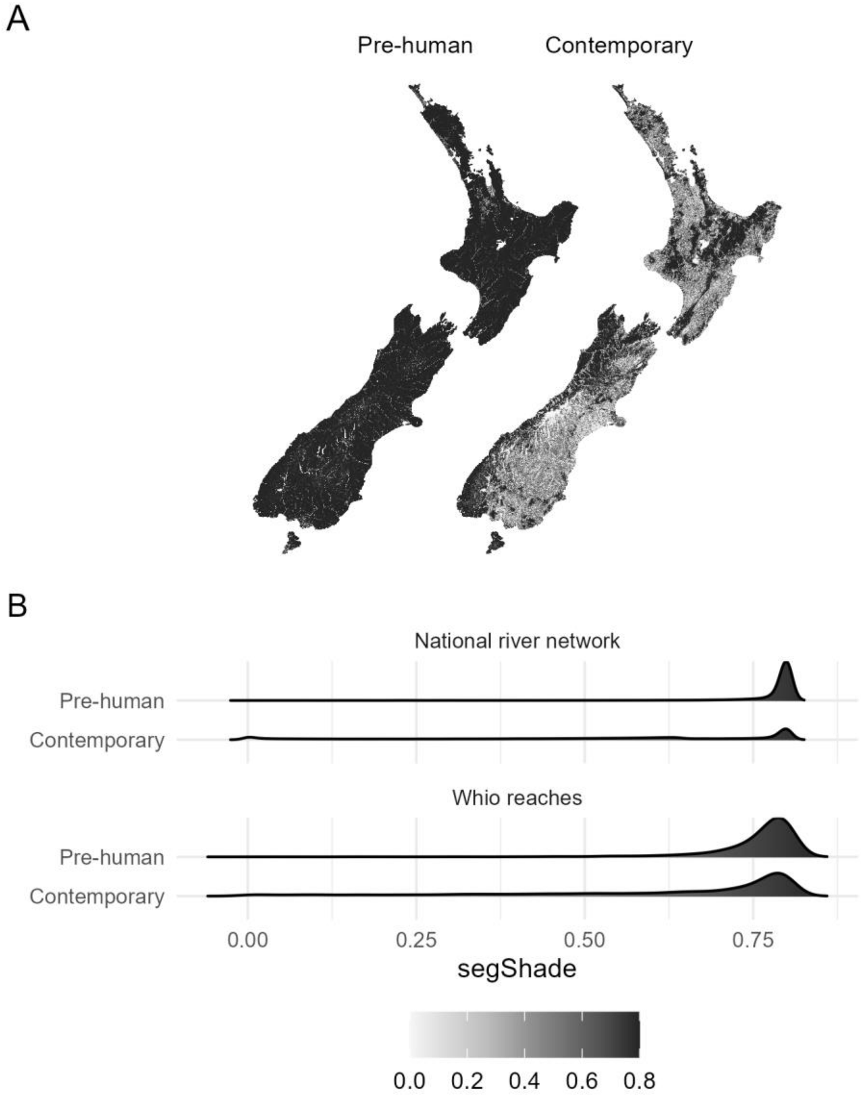

S1 Fig C. A) Spatial distribution and B) density plots of localised shade (*segShade*) across the national river network and at reaches where whio are known to occur. These data were used to predict the potential pre-human and potential contemporary distribution of whio, respectively. See Table 1 for predictor description and units. Data were obtained from the Freshwater Ecosystems of New Zealand (FENZ) geodatabase (republished under a CC BY license, with permission from Department of Conservation, original copyright 2010).

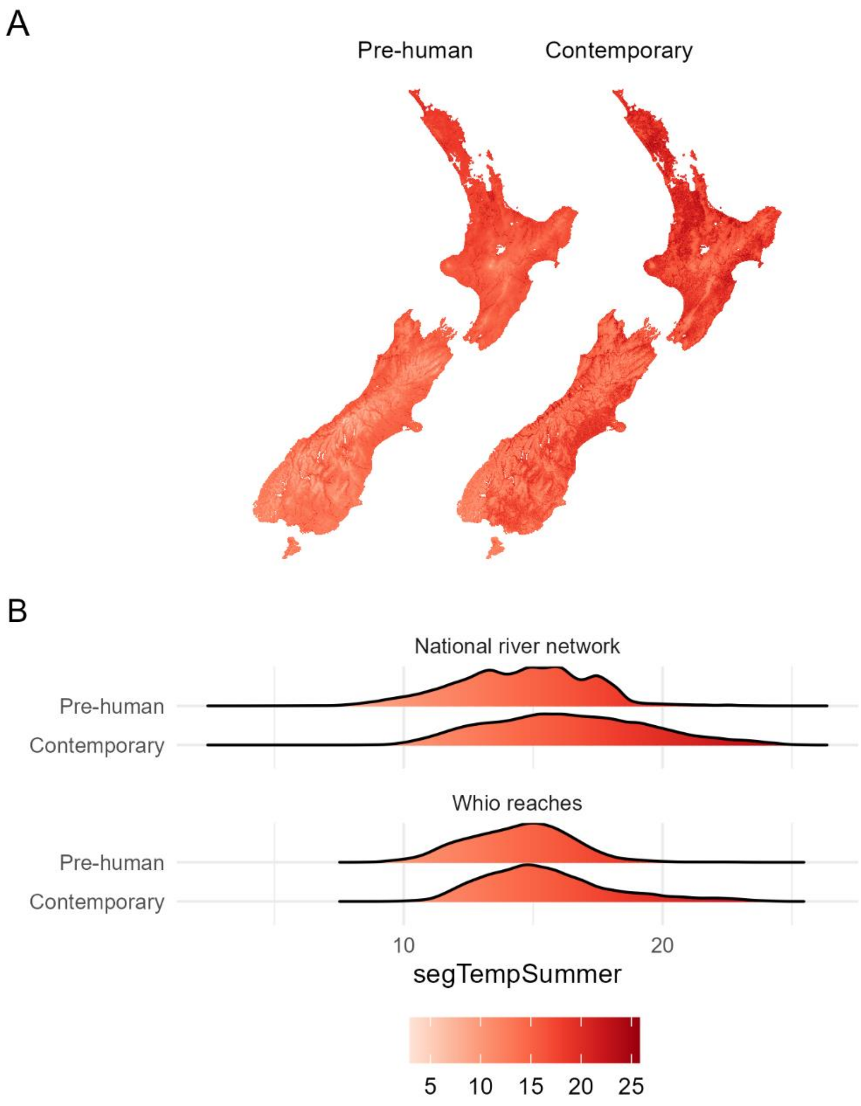

S1 Fig D. A) Spatial distribution and B) density plots of pre-human and contemporary summer air temperature (*segTempSummer*) across the national river network and at reaches where whio are known to occur. These data were used to predict the potential pre-human and potential contemporary distribution of whio, respectively. See Table 1 for predictor description and units. Data were obtained from the Freshwater Ecosystems of New Zealand (FENZ) geodatabase (republished under a CC BY license, with permission from Department of Conservation, original copyright 2010).

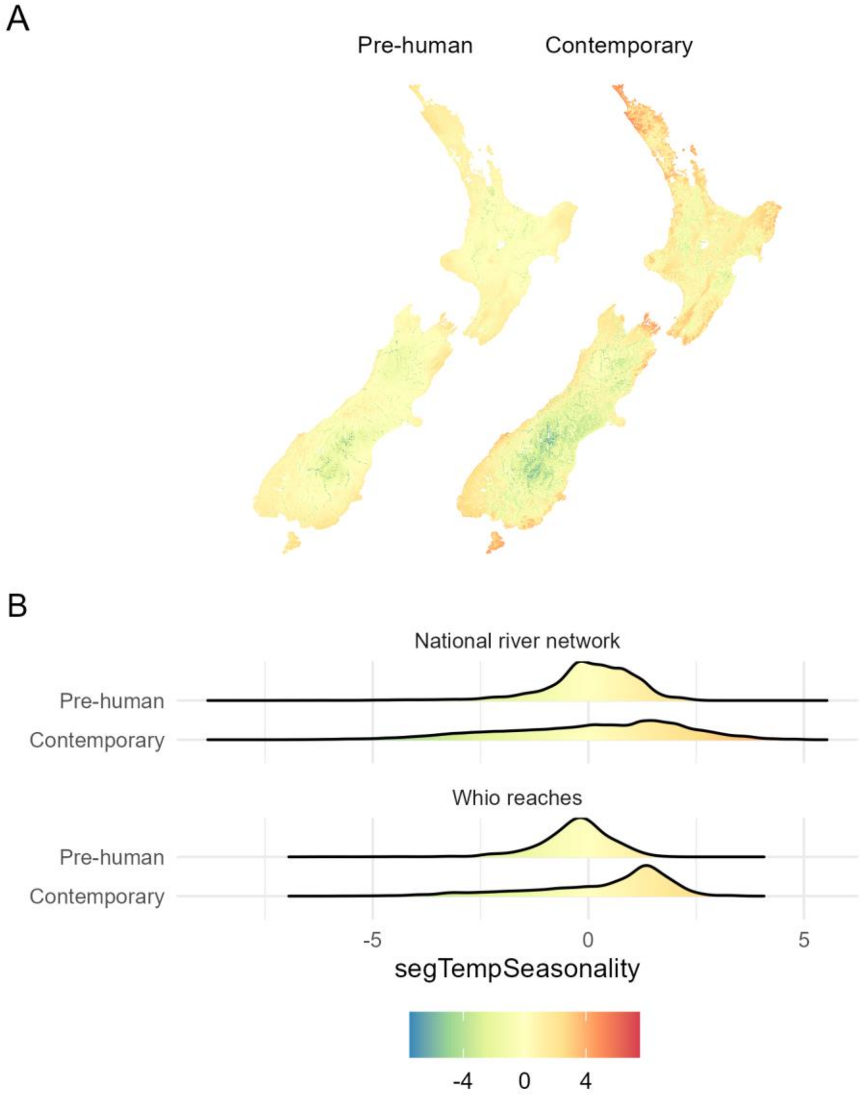

S1 Fig E. A) Spatial distribution and B) density plots of pre-human and contemporary winter air temperature normalized with respect to summer air temperature (*segTempSeasonality*) across the national river network and at reaches where whio are known to occur. These data were used to predict the potential pre-human and potential contemporary distribution of whio, respectively. See Table 1 for predictor description and units. Data were obtained from the Freshwater Ecosystems of New Zealand (FENZ) geodatabase (republished under a CC BY license, with permission from Department of Conservation, original copyright 2010).
