# Supplementary material for: Quantifying the relative contributions of habitat modification and mammalian predators on landscape-scale declines of a threatened river specialist duck
Source: PLoS One. 2022 Dec 30;17(12):e0277820. doi: 10.1371/journal.pone.0277820 (PMC9803212; doi:10.1371/journal.pone.0277820)
Supplement: S2 File — (PDF) [file pone.0277820.s002.pdf]

S2 File. ODMAP (Overview, Data, Model, Assessment and Prediction) protocol describing the species distribution modelling approach used in this analysis using a standardized reporting framework.

Quantifying the relative contributions of habitat modification and introduced predators on landscape-scale declines of a threatened river specialist duck

– ODMAP Protocol –

Amy Whitehead, John Leathwick, Doug Booker, Angus McIntosh

2022-05-20

Overview

Authorship

Contact: amy.whitehead@niwa.co.nz

Model objective

Model objective: Forecast and transfer

Target output: Relative change in suitable habitat between scenarios

Focal Taxon

Focal Taxon: Whio (*Hymenolaimus malacorhynchos*)

Location

Location: New Zealand

Scale of Analysis

Spatial extent: 166.4318, 178.5489, -47.2855, -34.39661 (xmin, xmax, ymin, ymax)

Spatial resolution: Watershed scale (0.46 ± 0.41 km2)

Temporal extent: 2020 and ~1200

Temporal resolution: N/A

Boundary: natural

Biodiversity data

Observation type: field survey, citizen science

Response data type: presence-only

Predictors

Predictor types: climatic, habitat, topographic

Hypotheses

Hypotheses: That the relative likelihood of occurrence will be higher in steeper, fast-flowing catchments that are dominated by a high proportion of upstream indigenous forest.

Assumptions

Model assumptions:

1) All relevant environmental drivers are included in the model,
2) the species' observed distribution is in equilibrium with the environment,
3) the entire realised niche is encompassed by the data,
4) the correlation structure between predictors does not change between the source and target landscapes/scenarios,
5) that unoccupied reaches represent true absences, and
6) the observed range contractions have been caused by habitat modification and the presence of introduced mammalian predators.

Algorithms

Modelling techniques: brt

Model averaging: None

Workflow

Model workflow: We developed models of the relative likelihood occurrence for whio using boosted regression trees, with model performance assessed by making predictions at sites that were withheld from model development. We ran 200 model simulations, each fitted to a bootstrapped re-sample of the original dataset that allowed us to calculate uncertainty in the outputs. These bootstrapped simulations used the same model structure but randomly withheld 20 % of the data from the full dataset. The withheld data were used to assess model performance using two metrics: the predictive deviance, and the discrimination between presences and absences as measured by the area under the receiver operator characteristic curve.

We generated spatial predictions across the New Zealand digital river network for each simulation under two different scenarios: a potential contemporary scenario where we predicted to the contemporary environment, and a potential pre-human scenario where we predicted to the environment prior to human arrival in New Zealand (~1200AD).

Software

Software: R (Verson 3.3.3, R Core Team 2017), dismo (version 1.1-4), dsmextra (version 1.1.5)

Data

Biodiversity data

Taxon names: *Hymenolaimus malacorhynchos*

Taxonomic reference system: The Integrated Taxonomic Information System

Ecological level: species

Data sources: Supplied by the New Zealand Department of Conservation (2017)

Sampling design: None

Sample size: 17133 observations

Clipping: New Zealand

Scaling: Observations thinned to 1 record per reach on the New Zealand digital river network, resulting in 5,439 reaches identified as occupied by whio.

Cleaning: Removed a small number of observations that did not occur close to a river - these were assumed to be errors in location recording.

Absence data: None

Background data: We randomly selected 5,439 reaches from a geographically constrained area based on a kernel density plot of whio presences to use as background points where whio were assumed to be absent.

Errors and biases: Misidentification of whio is unlikely as they are a very distinctive species. No information was available on detection probability or sampling bias.

Data partitioning

Training data: 80% of presences and background data were selected at random without resampling for each bootstrapped model run

Validation data: Internal cross-validation method used by gbm.fixed

Test data: Data not used in model development (i.e., 20% of presences and background data) for each bootstrapped model run

Predictor variables

Predictor variables: segTempSummer, segTempSeasonality, segSinuosity, segSlop, segSlopeCatchment, Feb, FRE3, segFlowStability, MeanFlow, Order, WidthQ50, segIndigenousForest, usIndigenousForest, segShade, segSediment, segGravelCobble

Descriptions of each predictor are provided in Table 1 of paper.

segIndigenousForest, usIndigenousForest, segShade, segTempSummer and segTempSeasonality were replaced with estimates of values prior to human arrival in New Zealand for the potential pre-human scenario

Data sources: Leathwick, J.R., West, D.W., Gerbeaux, P., Kelly, D., Robertson, H.A., Brown, D., Chadderton, W.L., Ausseil, A.-G.E. (2010) Freshwater Ecosystems of New Zealand (FENZ) Geodatabase: User Guide. Department of Conservation.

Spatial extent: 166.4318, -47.2855, 178.5489, -34.39661 (xmin, xmax, ymin, ymax)

Spatial resolution: Watershed scale (0.46 ± 0.41 km2) of the New Zealand digital river network

Coordinate reference system: EPSG: 2193

Temporal extent: 2010

Temporal resolution: N/A

Data processing: Three predictors (MeanFlow, WidthQ50, segSinuosity) were log-transformed prior to analysis.

Errors and biases: None

Dimension reduction: None

Transfer data

Data sources: Leathwick, J.R., West, D.W., Gerbeaux, P., Kelly, D., Robertson, H.A., Brown, D., Chadderton, W.L., Ausseil, A.-G.E. (2010) Freshwater Ecosystems of New Zealand (FENZ) Geodatabase: User Guide. Department of Conservation.

Spatial extent: 166.4318, -47.2855, 178.5489, -34.39661 (xmin, xmax, ymin, ymax)

Spatial resolution: Watershed scale (0.46 ± 0.41 km2) of the New Zealand digital river network

Temporal extent: Potential contemporary scenario: 2010 Potential pre-human scenario: ~ 1200AD

Temporal resolution: N/A

Models and scenarios: Potential contemporary scenario: Made predictions across the entire New Zealand river network based on contemporary habitat conditions. Potential pre-human scenario: Made predictions across entire New Zealand river network based on estimated environmental conditions prior to human arrival in New Zealand.

Data processing: Three predictors (MeanFlow, WidthQ50, segSinuosity) were log-transformed prior to analysis.

Quantification of Novelty: Extrapolation Detection (exDet) Tool (Bouchet et al 2019)

Model

Variable pre-selection

Variable pre-selection: Based on previous research and ecological relevance

Multicollinearity

Multicollinearity: Multicollinearity was not explicitly dealt with as all predictors were deemed to be ecologically important

Model settings

brt: distribution (bernoulli), nTrees (2000), interactionDepth (5), shrinkage (0.01), bagFraction (0.5), trainFraction (0.8)

Model settings (extrapolation): None, although BRT models inherently cannot extrapolate beyond the range of the input data

Model estimates

Coefficients: N/A as BRT models do not produce coefficients

Parameter uncertainty: 200 bootstrapped models based on resampling

Variable importance: The method implemented in the gbm package which underpins dismo

Model selection - model averaging - ensembles

Model selection: N/A

Model averaging: N/A

Model ensembles: N/A

Analysis and Correction of non-independence

Spatial autocorrelation: None

Temporal autocorrelation: N/A

Nested data: None

Threshold selection

Threshold selection: N/A

Assessment

Performance statistics

Performance on training data: AUC, Predictive deviance

Performance on validation data: AUC, Predictive deviance

Performance on test data: N/A

Plausibility check

Response shapes: Partial response plots

Expert judgement: Map display

Prediction

Prediction output

Prediction unit: Individual watersheds on the New Zealand digital river network

Post-processing: None

Uncertainty quantification

Algorithmic uncertainty: N/A

Input data uncertainty: N/A

Parameter uncertainty: Calculated prediction uncertainty for each scenario as the coefficient of variation at each watershed based on predictions from 200 bootstrapped BRT models

Scenario uncertainty: Unknown

Novel environments: Visualised novel environments in map display
