# Supplementary material for: Quantifying the relative contributions of habitat modification and mammalian predators on landscape-scale declines of a threatened river specialist duck
Source: PLoS One. 2022 Dec 30;17(12):e0277820. doi: 10.1371/journal.pone.0277820 (PMC9803212; doi:10.1371/journal.pone.0277820)
Supplement: S1 Table — Performance of the full model was assessed using 10-fold cross-validation, while performance of the 200 bootstrapped models was assessed by comparing predictions to data withheld during each simulation. (PDF) [file pone.0277820.s003.pdf]

S3 Table. Mean (± se) predictive performance of a boosted regression tree (BRT) model predicting the relative likelihood of occurrence (RLO) for whio across New Zealand. Performance of the full model was assessed using 10-fold cross-validation, while performance of the 200 bootstrapped models was assessed by comparing predictions to data withheld during each simulation.

**Model performance**	**Percentage deviance explained**	**Area under the receiver operator curve (AUC)**
Full model	69.4 ± 1.0	0.925 ± 0.002
Bootstrapped models	69.2 ± 0.2	0.925 ± 0.000
