# Supplementary figures and images for: Quantifying the relative contributions of habitat modification and mammalian predators on landscape-scale declines of a threatened river specialist duck
Source: PLoS One. 2022 Dec 30;17(12):e0277820. doi: 10.1371/journal.pone.0277820 (PMC9803212; doi:10.1371/journal.pone.0277820)

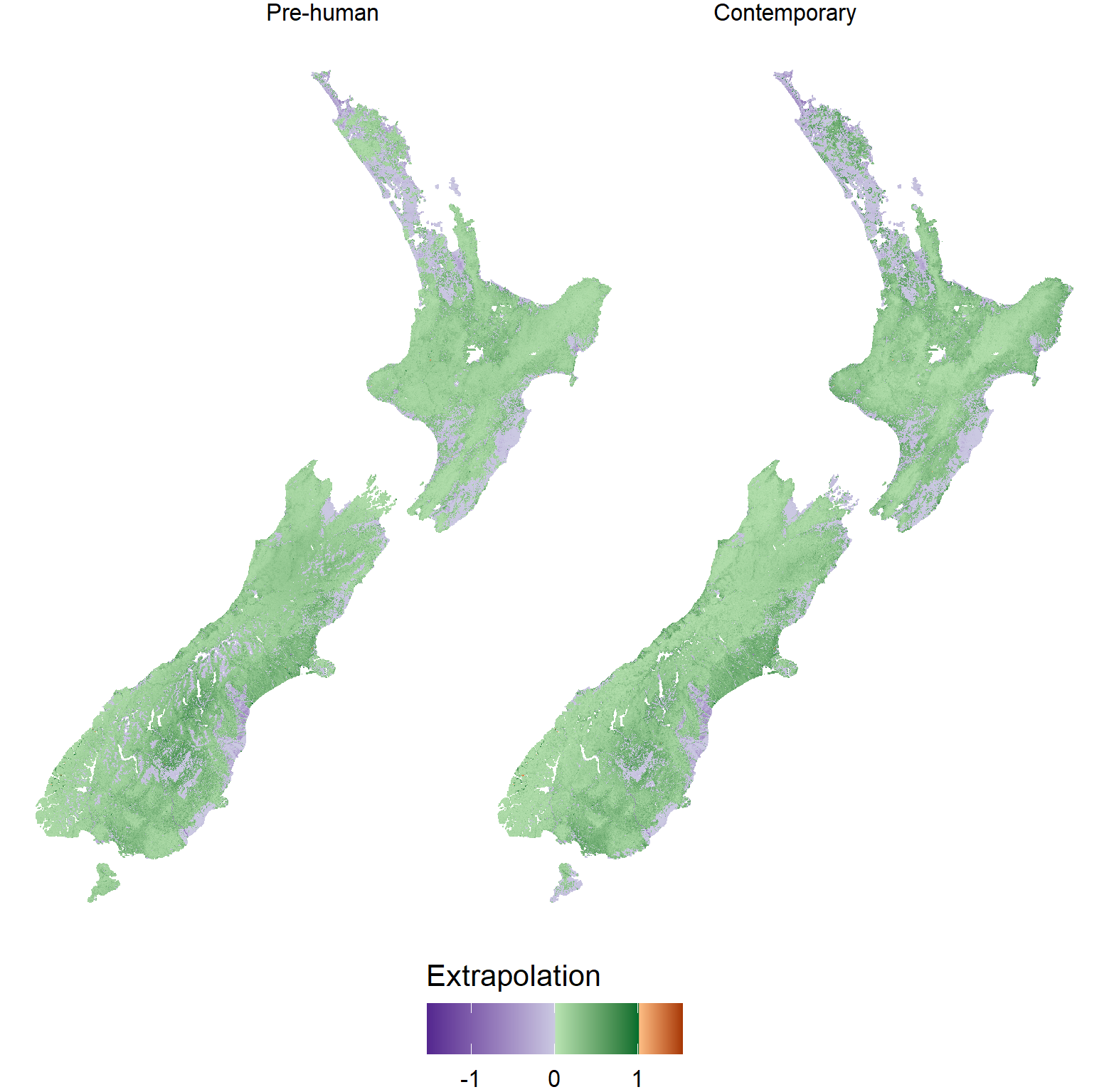

Supplement: S1 Fig — Negative values (purple) indicate areas with at least one predictor outside the univariate range of the whio occurrences (Table 1). Values greater that one (orange) indicates areas that are within the univariate coverage of the whio occurrences but represent non-analogous predictor combinations. Values ranging 0 to 1 (green) are similar to the whio occurrences as they both fall within the range of predictors and capture the same predictor combinations. (TIF) [file pone.0277820.s004.tif]

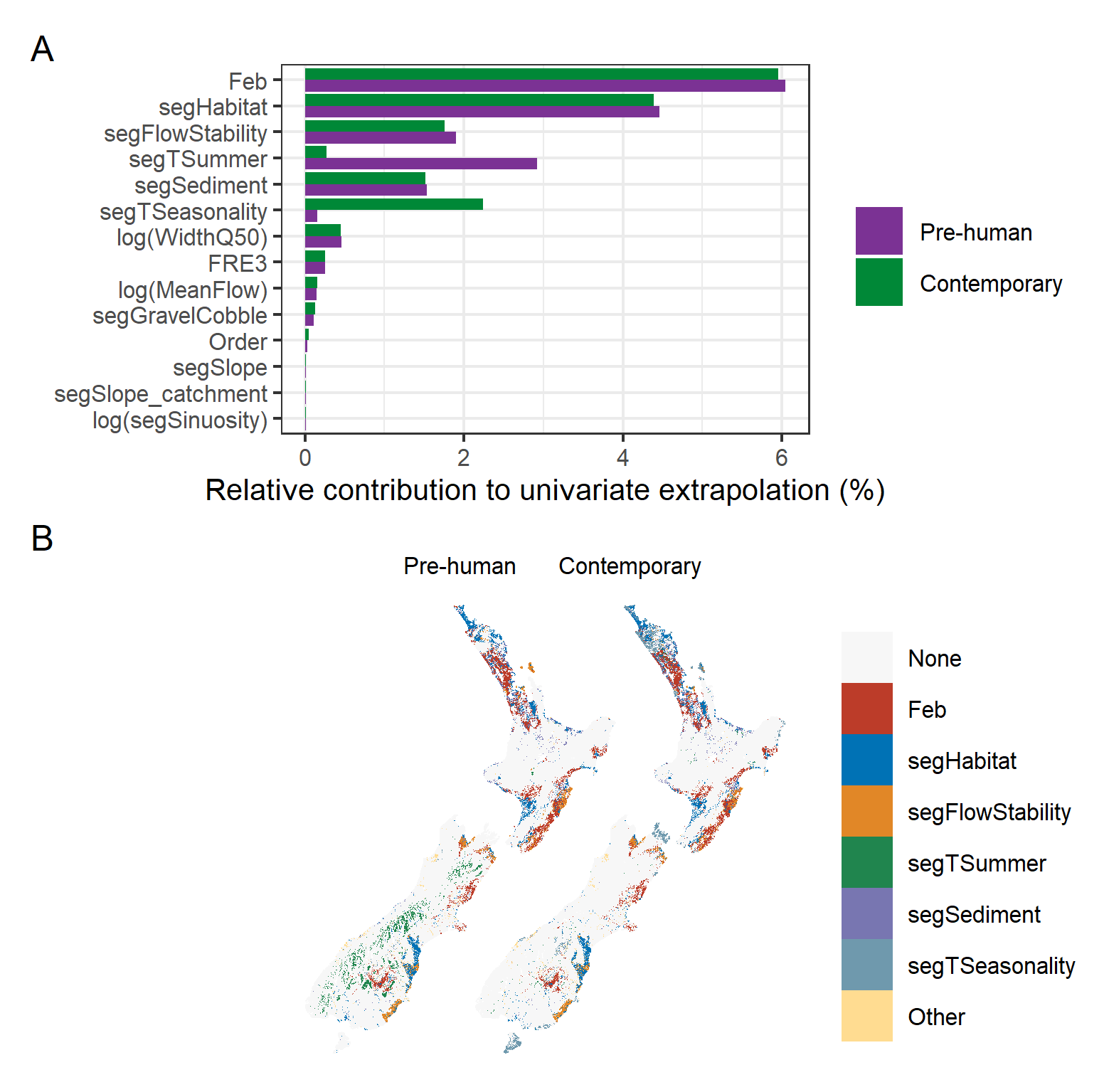

Supplement: S2 Fig — A) The most influential univariate predictors affecting extrapolation within the pre-human and contemporary environments with respect to whio occurrences. B) The spatial distribution of the most dissimilar predictor relative to whio occurrences at a given location. Predictors contributing less than 1.5% to univariate extrapolation have been combined into Other, while None reflects areas where no predictor is outside the coverage of the whio occurrences or with non-analogous combinations. Note that segIndigenousForest and usIndigenousForest do not contribute to extrapolation as whio occur across the full range of values in the environment (Table 1). See Table 1 for predictor description and units. (TIF) [file pone.0277820.s005.tif]

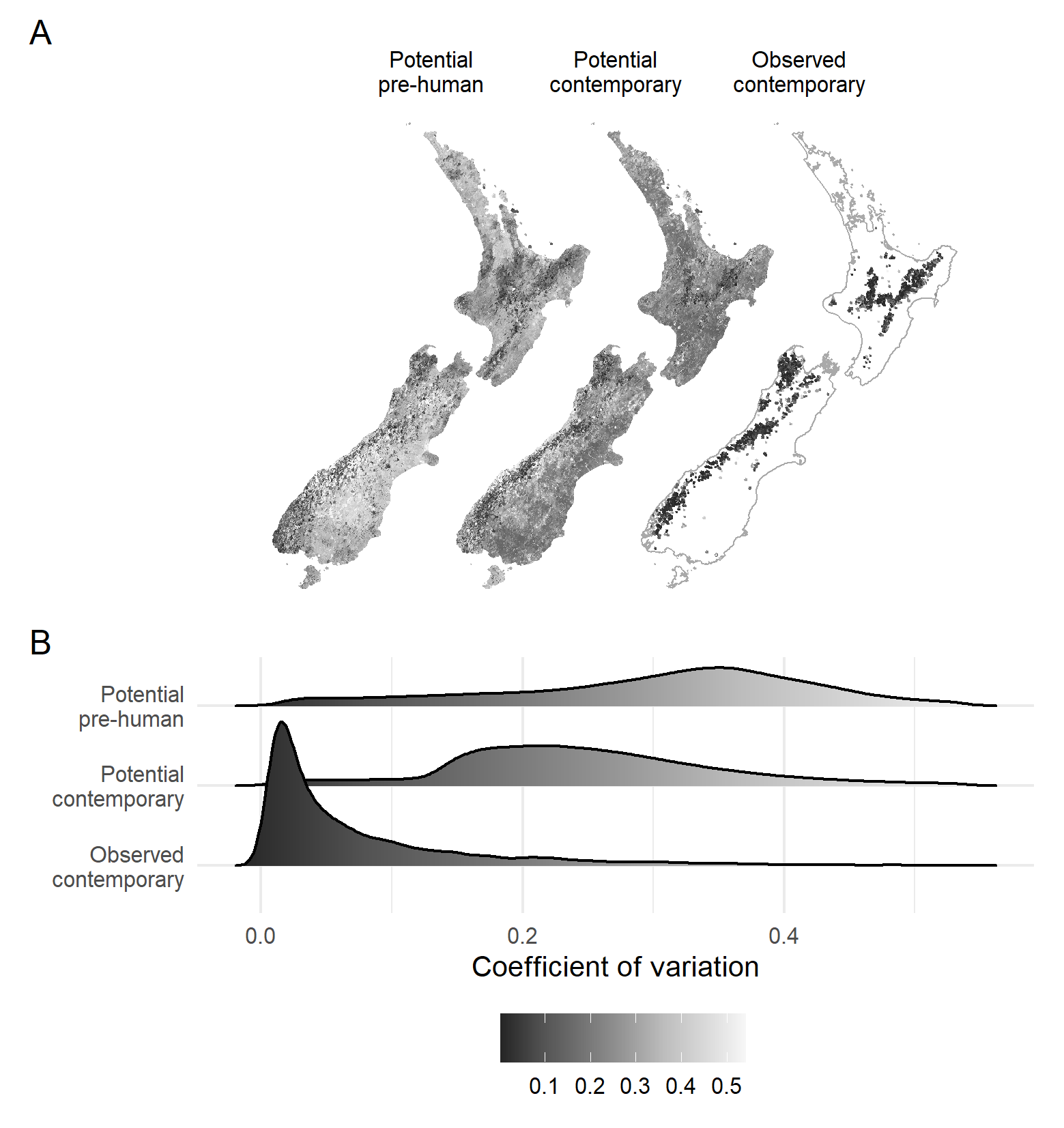

Supplement: S3 Fig — A) Spatial distribution and B) density plots of uncertainty (coefficient of variation) in the relative likelihood of occurrence (RLO) prior to human arrival in New Zealand (potential pre-human), in contemporary habitat with the exclusion of predators (potential contemporary) and the observed distribution between 1979 and 2016 (observed contemporary). The potential values are based on predictions from 200 bootstrapped simulations of a boosted regression tree model, while the observed values represent the uncertainty from the potential contemporary models at all reaches where whio were known to occur between 1979 and 2016. The top 2.5% of uncertainty values have been removed to add visual interpretation of the data. (TIF) [file pone.0277820.s006.tif]

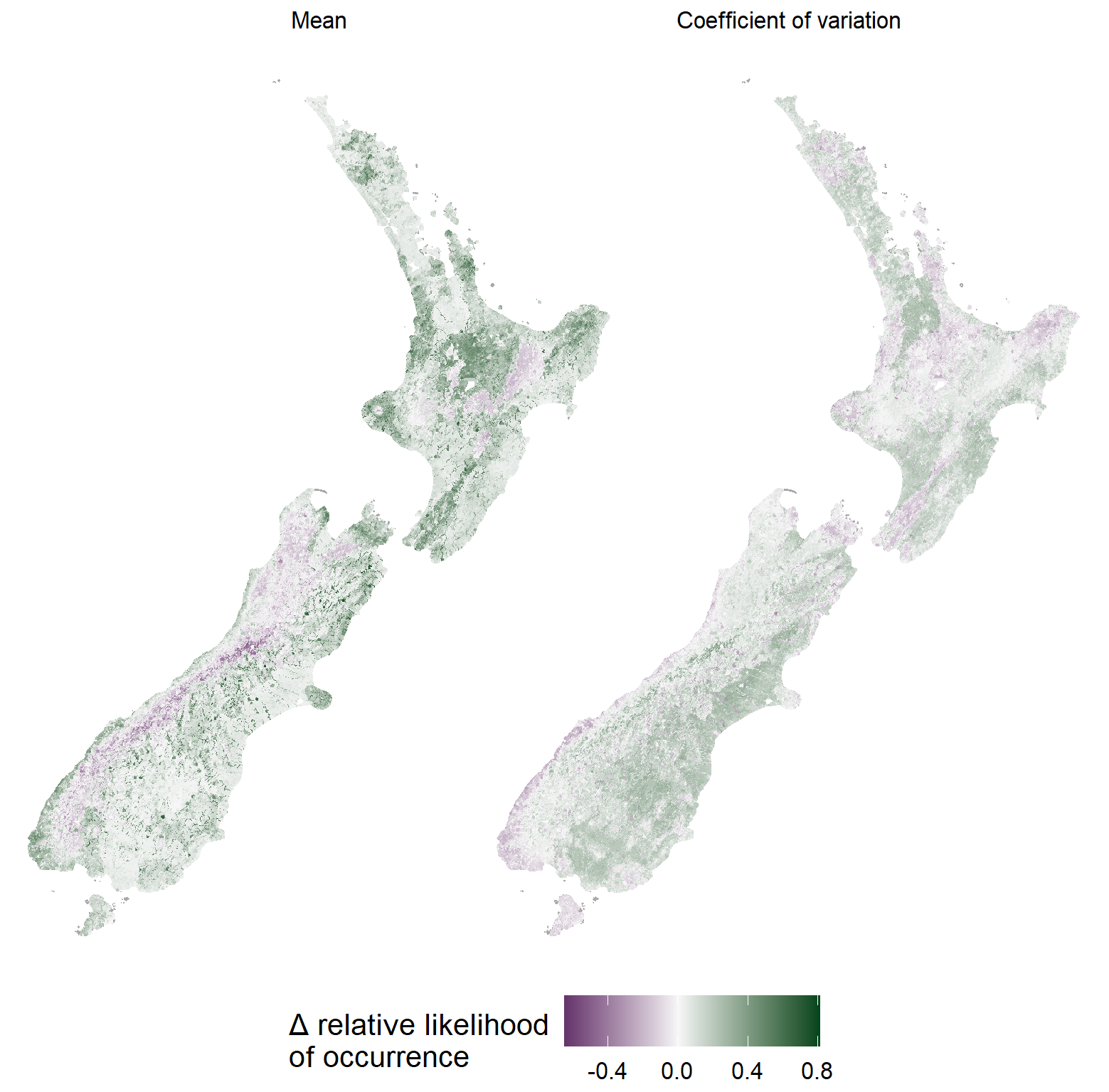

Supplement: S4 Fig — Negative values (purple) indicate areas with lower pre-human values, while positive values (green) occur in areas with higher pre-human values. (TIF) [file pone.0277820.s007.tif]
